# Increased frequencies of Th17 in the peripheral blood of patients with chronic lymphocytic leukemia: A one year follow-up

**DOI:** 10.12669/pjms.305.5252

**Published:** 2014

**Authors:** Dijiao Tang, Qian Niu, Nenggang Jiang, Jingjing Li, Qin Zheng, Yongqian Jia

**Affiliations:** 1 Dijiao Tang, Department of Laboratory Medicine, West China Hospital, Sichuan University, Chengdu, P. R. China.; 2 Qian Niu, Department of Laboratory Medicine, West China Hospital, Sichuan University, Chengdu, P. R. China.; 3 Nenggang Jiang, Department of Laboratory Medicine, West China Hospital, Sichuan University, Chengdu, P. R. China.; 4 Jingjing Li, Department of Laboratory Medicine, West China Hospital, Sichuan University, Chengdu, P. R. China.; 5 Qin Zheng, Department of Laboratory Medicine, West China Hospital, Sichuan University, Chengdu, P. R. China.; 6 Yongqian Jia, Department of Hematology, West China Hospital, Sichuan University, Chengdu, P. R. China. Department of Laboratory Medicine, West China Hospital, Sichuan University, Chengdu, P. R. China.

**Keywords:** Th17 cells, Treg cells, CLL, Follow-up

## Abstract

***Objective*** ***:*** In this study, we aimed to investigate changes of peripheral Th17 and Treg cells frequencies in the newly-diagnosed Chronic Lymphocytic Leukemia (CLL) patients for 12 months.

***Methods*** ***:*** In this research, 50 CLL patients were enrolled. Circulating Th1, Th17 cells and CD4+CD25+Foxp3+Treg cells were analyzed by flow cytometry. Plasma levels of related cytokines were detected by enzyme-linked immuno sorbent assay (ELISA). The study was carried out from January 2012 to October 2013 at Department of Laboratory Medicine, West China Hospital, Sichuan University, Chengdu, P.R. China.

***Results:*** Compared with healthy controls, Th17 cells related cytokines were significantly increased in CLL patients, while Treg cells related cytokines were significantly lowered. In the follow-up, we found that the frequency of Treg cells was irregular, while the frequency of Th17 cells was gradually decreased.

***Conclusion:*** Our study suggested that Th17 cells may play important role in the immune regulation of CLL, and may become a new target in CLL therapy.

## INTRODUCTION

Chronic Lymphocytic Leukemia (CLL) is a low-grade lymphoproliferative tumor, which is characterized by monoclonal B lymphocytes accumulation, apoptosis inhibition as well as infiltration of peripheral blood, bone marrow and lymph nodes.^1^ A large number of evidences suggested that the CD4^+ ^T cell-mediated autoimmune regulator imbalance may play a key role in the pathogenesis and development of CLL.^2^^,^^3^

CD4^+^ T helper cells (Th17 cell) and CD4^+^CD25^+^ Foxp3^+^ regulatory T cells (Treg cell) are two novel subsets of CD4^+^ T cell. Th17 cell is an important mediator of cancer, chronic inflammation and autoimmune diseases through secretion of pro-inflammation cytokines, such as IL-17A, IL-17F, IL-22, IL-21 and IFN-γ.^4^^,^^5^ Treg cell plays a role in anti-inflammatory and maintain autoimmune tolerance, which also shows negative immuno-regulatory function via cell-contact inhibition and secretion of inhibitory cytokines (such as IL-10, TGF-β).^6^ Both of Th17 and Treg cells can be activated by autoimmune or inflammation-mediated immune response.^7^ Lots of studies have shown that Th17/Treg imbalance played an important role in autoimmune diseases and tumorigenesis.^8^^,^^9^ However, until now, there are no follow-up on cell level of CLL in China. Our primary results showed that the frequency of Th17 cells was elevated and the frequency of Treg cells was reduced in the peripheral blood of CLL patients.^10^

In this study, we evaluated the cytokines secreted by Th17 and Treg cells in the peripheral blood of CLL patients. Furthermore, we followed up the frequencies of Treg and Th17 cells in patients with CLL (Rai stage III and IV, Rai stage: cytologic staging system for chronic lymphocytic leukemia, which divides it into low (0), intermediate (I and II), and high-risk stages (III and IV).

## METHODS

***Patients:*** From January 2012 to October 2013, Peripheral blood from 20 healthy individuals (16 males; 4 females; mean age 59.4±15.8 years) and 50 patients with CLL (38 males; 12 females; mean age 61.7±12.3 years) were obtained following approval by the Ethics Committee of the Chinese Human Genome and the Ethics Committee of West China Hospital, and informed consents were obtained from all participants. All peripheral blood samples from healthy donors (controls) and patients were anti-coagulated with heparin. Diagnostic criteria for CLL were based on WHO Classification of Tumours of Haematopoietic and Lymphoid Tissues, and CLL remission criteria were based on the National Cancer Institute-sponsored Working Group (NCI-WG) on Chronic Lymphocytic Leukemia (including PR and CR). Staging was performed according to the Rai classification for CLL. Patients’ characteristics of all patients are summarized in Table-I. Twenty newly-diagnosed patients (Rai stage III-IV) were followed up at the time of 3 months (M3), 6 months (M6) and 12 months (M12) after the treatment with Chlorambucil (Gloxo SmithKline, UK), and 10 newly-diagnosed Rai stage I-II and 20 remission patients were excluded in the follow-up.

***Antibodies: ***Cell phenotype of T cells was defined by multicolor flow cytometry. All the antibodies, including peridinin chlorophyll protein (PerCP)-conjugated CD3; fluorescein isothiocyanate (FITC)-conjugated CD8; phycoerythrin (PE) - conjugated IL-17A; allophycocyanin (APC) - conjugated IFNγ; PerCP-CD3/FITC-CD4/PE-CD8 and the corresponding isotype control antibodies were from Becton Dickinson Biosciences (San Diego, USA). Human Treg Staining Kit (including Fixation /Permeabilization) was from eBioscience (San Diego, California, USA). Cells were stained according to the manufacturer’s recommendations.

***Cell preparation: ***For analysis of Th17, 500 μl of whole blood sample was cultured in complete culture medium (RPMI 1640 supplemented with 10% heat-inactivated fetal calf serum) for 4 h, in the presence of phorbol myristate acetate (PMA, PMA is used to stimulate the intracellular cytokine production together with ionomycin, 50 ng/ml, Sigma, USA), ionomycin (1 μg/ml, Sigma, USA) and monensin (a polyether antibiotic, 1 μg/ml, BD, USA). The incubators were set at 37°C, 5% CO_2_. For analysis of Treg, 50 μl of whole blood sample was aliquoted into a tube for further staining.

***Surface and intracellular staining: ***For Th17 analysis, 70 μl of stimulated whole blood was incubated with PerCP-conjugated CD3 and FITC-conjugated CD8 at 4 °C for 30 min. (Because of reduced CD4 expression after PMA stimulation in human samples, we focused on CD3+CD8-IL-17+ T cells as Th17 cells) For Treg analysis, 50 μl of whole blood without stimulation was incubated with FITC-CD4/APC-CD25 cocktail at 4°C for 30 minutes. After the surface staining, the samples were stained with phycoerythrin (PE)-conjugated IL-17A for Th17 detection or PE-conjugated Foxp3 for Treg detection after fixation and permeabilization according to the manufacturer's instructions (Human Treg Staining Kit, eBioscience). Isotype controls were given to enable correct compensation and confirm antibody specificity. Stained cells were run on a FACSCalibur cytometer (BD Bioscience), and the data were analysed using FACSDiva software (BD Bioscience).

***Enzyme-linked immunosorbent assay (ELISA): ***For all patients and control subjects, a 3-mL fasting blood sample was drawn into a BD Vacutainer tube containing heparin, plasma was obtained after centrifugation and stored at −20°C for the measurement of the cytokines. The plasma concentrations of IL-17, IL-23, IL-6, IL-10 and TGF-β1 were measured by ELISA, following the manufacturer’s instructions, IL-17, IL-23, IL-10 and TGF-β1 ELISA kits were from Bender MedSystems, Burlingame, USA. IL-6 kit was from Neobioscience, Shenzhen, China. All samples were measured in duplicate.

***Statistical analysis: ***Values are expressed as mean±SD or median (range) in the tables and figures. Summary statistics, such as percentages, medians, means, SDs, were used to describe the patients’ baseline characteristics. Pairwise comparisons between groups were conducted using Student *t *tests and further verified by the Wilcoxon tests based on ranks. When multiple groups are present, the analysis of variance models and the analysis of variance F tests were used to evaluate the overall differences among these groups. Statistical significance was defined as p <0.05. Data were analyzed using SPSS 16.0 software.

## RESULTS

***Plasma concentrations of cytokines in patients with CLL: ***As shown in Table-II, plasma concentrations of IL-17A, IL-23 and IL-6 in the untreated group (IL-17: 3.23 (1.33-17.9) pg/ml; IL-23: 78.06 (0.79-236.73) pg/ml; IL-6: 9.28 (0.42-511.24) pg/ml) increased significantly compared to the healthy control group (IL-17: 0.71 (0.39-9.94) pg/ml; IL-23: 18.03 (13.69-27.16) pg/ml; IL-6: 0.77 (0.53-1.06) pg/ml; p<0.05). Plasma concentrations of IL-10 and TGF-β1 in the untreated group (3.16 (0.02-30.85 pg/ml and 1.33 (0.049-8.87) ng/ml, respectively) decreased significantly compared to the healthy control group (9.83 (7.30-11.31) pg/ml and 24.56 (13.70-31.60) ng/ml, respectively (p<0.05).

**Fig.1:**
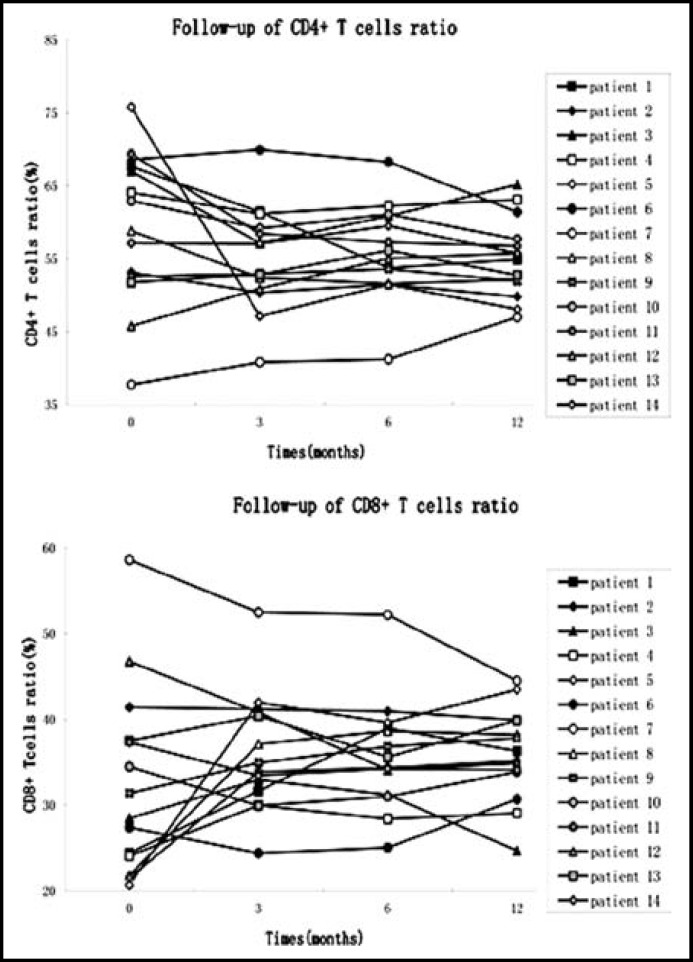
Follow-up of CD4+ and CD8+ T cells ratios. Both of the results showed no statistical differences among newly diagnosis and 3, 6, 12 months (M3, M6, M12). [CD4+ T cells: M0 vs M3, p=0.084; M0 vs M6, p=0.245; M0 vs M12, p=0.074; M3 vs M6, p=0.158; M3 vs M12, p=0.730; M6 vs M12, p=0.397. CD8+ T cells: M0 vs M3, p=0.272; M0 vs M6, p=0.300; M0 vs M12, p=0.245; M3 vs M6, p=0.826; M3 vs M12, p=0.778; M6 vs M12, p=0.638.]

**Fig.2:**
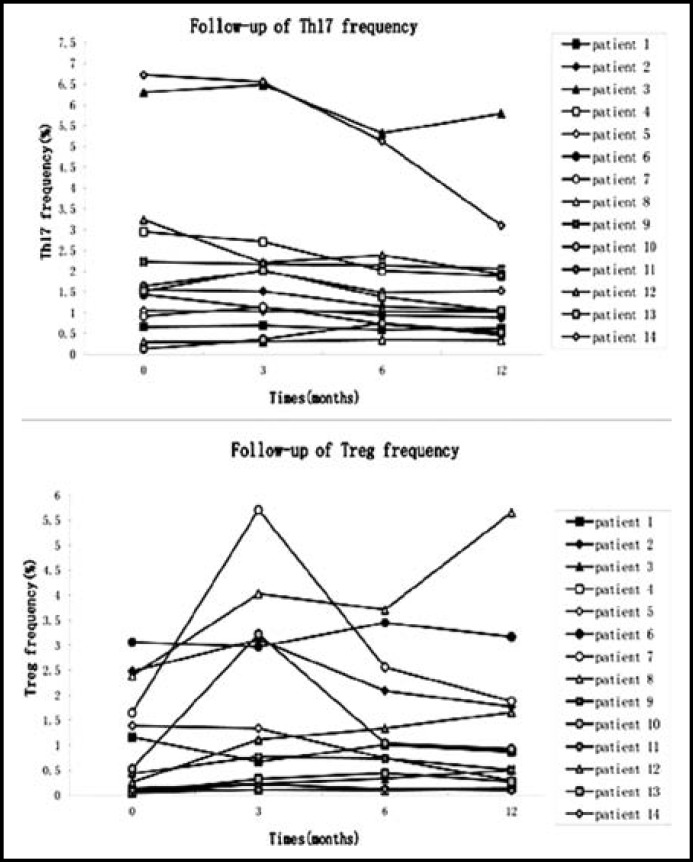
Follow-up of Th17 and Treg frequencies. Th17 frequency showed a continuous decline from M3 to M12. The changes of Treg frequency were irregular. The Treg frequency was significantly elevated in M3, but showed no statistical significance in M6 and M12.[Th17 cells: M0 vs M3, p=0.925; M0 vs M6, p=0.011; M0 vs M12, p=0.008; M3 vs M6, p=0.026; M3 vs M12, p=0.004; M6 vs M12, p=0.044. Treg cells: M0 vs M3, p=0.020; M0 vs M6, p=0.069; M0 vs M12, p=0.198; M3 vs M6, p=0.379; M3 vs M12, p=0.490; M6 vs M12, p=0.258.]

**Fig.3:**
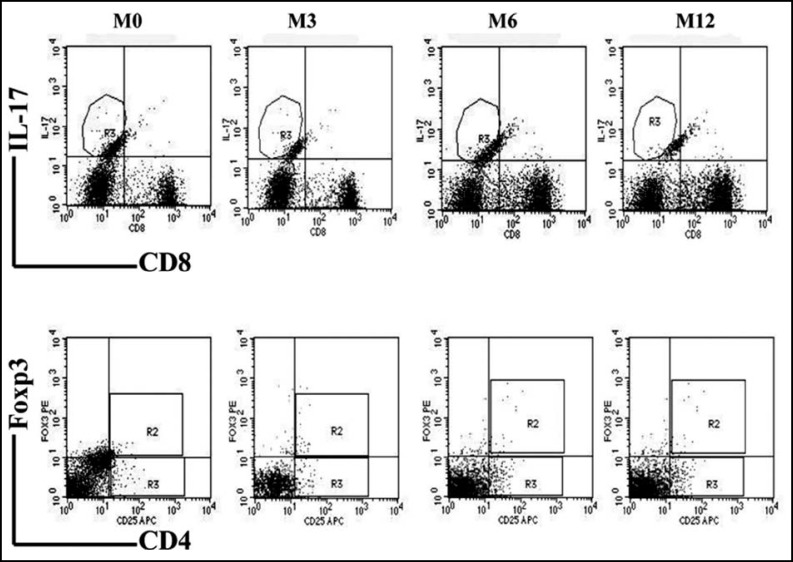
Decreasing frequencies of Th17 cells in patients with CLL after treatment. The changes of Treg frequency were irregular. Plots in intern box represented flow cytometric analysis at M0, M3, M6 and M12 of Th17 (CD8-IL17+) cells(Top panels) and Treg(CD4+CD25+Foxp3+) cells(Bottom panels) from a single subject (patient 12) who had the typical changes in the follow-up

***Change in Treg and Th17 cell numbers in Rai III and IV CLL patients at 12 month follow-up: ***Twenty CLL patients at Rai stage III and IV were followed for one year, at which time 14 patients continued in the study. Five patients dropped out of the study to receive treatment in other hospitals, and one patient could no longer be contacted. We tested lymphocyte numbers, CD4+ and CD8+ T cells ratios (Fig.1), and Treg and Th17 cell frequencies (Fig.2) in patients after 3, 6, and 12 months (M3, M6, M12) of constant standard treatment with chlorambucil (Gloxo SmithKline, UK). Flow cytometric analysis of a typical patient is shown in Fig.3.

After treatment for 3 months, the number of lymphocytes was significantly decreased compared to pretreatment numbers (12.70±10.20 ×10^9^ vs. 70.33±101.83×10^9^ , p<0.001), and Treg frequency was significantly elevated (1.71±1.78% vs. 0.98±1.05%, p<0.05). There was no significant difference between patients and control subjects in CD4+ T cell ratio (59.40±10.40 vs. 55.10±7.11), CD8+ T cell ratio (32.62±10.88 vs. 36.05±6.94), or Th17 cell frequency (2.19±2.04% vs. 2.16±1.98%).

After treatment for 6 months, a further reduction was observed in lymphocyte number (10.33±8.90 ×10^9^ vs. 12.70±10.20 ×10^9^, p<0.01) and Th17 cell frequency (1.81±1.56% vs. 2.16±1.98%, p<0.05), compared to 3 months earlier. No significant difference was observed in the CD4+ T cell ratio (55.10±7.11 vs. 55.87±6.47) or CD8+ T cell ratio (36.05±6.94 vs. 35.81±6.51). Irregular changes in Treg frequency did not achieve statistical significance (1.71±1.78% vs. 1.27±1.22%).

After 12 months treatment, an additional decline was observed in lymphocyte number (12.70±10.20 ×10^9 ^vs .7.84±8.14×10^9^ p<0.05) and Th17 cell frequency (1.81±1.56% vs. 1.59±1.43%, p<0.05), compared to six months treatment. There was no significant change in CD4+ T cell ratio (55.87±6.47 vs. 55.27±5.43), CD8+ T cell ratio (35.81±6.51 vs. 35.94±5.41) or Treg cell frequency (1.27±1.22% vs. 1.28±1.54%).

## DISCUSSION

CLL is uncommon in China, with an incidence of only 1/20–1/30. In recent years, an increasing number of patients have been diagnosed with CLL, most of whom were evaluated at middle or late stage upon first visit. Several studies have shown significant degradation of T lymphocyte function and ratios in patients with B-cell CLL,^11^ but the underlying cellular and molecular mechanisms for these changes are still unclear.

**Table-I:** Patient demographics (%, mean±SD).

**Characteristics**	**Untreated CLL(n=30)**	**Remission CLL(n=20)**	**Control(n=20)**
White blood cell count×10^9^	63.89±83.67 (7.61-338.0)*	9.36±9.57(2.28-32.40) ▲	6.84±2.05(4.23-9.5)
Absolute lymphocyte count×10^9^	52.18±82.91(5.12-321.01)*	6.89±8.52(0.87-24.99)* ▲	1.83±0.75(1.12-3.00)
Hemoglobin (g/L)	111.61±27.26(59-154)*	108.92±38.57(38-161)*	148.60±14.71(117-169)
Plateletes×10^9^	107.14±67.73(23-262)*	118.25±57.99(35-244)*	180.00±52.17(102-286)
Rai clinical stage			
0-II	10	7	
III-IV	20	13	
LDH(IU/L)	334.00±317.37(133-1258)*	240.45±96.31(115-412) ▲	181.89±23.82(124-213)
CD38 expression (cut-off 20%)			
Positive	8	4	
Negative	22	16	

* p<0.05 *vs. *Control,

▲ p<0.05 *vs. *Untreated CLL.

**Table-II:** Plasma concentrations of cytokines in CLL patients and healthy controls [median (range)].

**Group**	***n***	**IL-17** **(pg/ml)**	**IL-23** **(pg/ml)**	**IL-6** **(pg/ml)**	**IL-10** **(pg/ml)**	**TGF-** **β1** **(ng/ml)**
Untreated CLL	30	3.23* (1.33-17.9)	78.06* (0.79-236.73)	9.28* (0.42-511.24)	3.16* (0.02-30.85)	1.33* (0.049-8.87)
Remission CLL	20	2.69* (1.91-17.9)	65.85* (1.86-215.68)	4.51*▲ (1.59-39.55)	3.23* (0.92-62.18)	12.61*▲ (0.212-45.49)
Control	20	0.71 (0.39-9.94)	18.03 (13.69-27.16)	0.77 (0.53-1.06)	9.83 (7.30-11.31)	24.56 (13.70-31.60)

* p<0.05 *vs. *Control,

▲ p<0.05 *vs. *Untreated CLL.

In our primary study, we found that the number of CD4+ T cells in the peripheral blood of CLL patients was elevated, and that therapy reduced the aberrant ratio of these cells.^10^ However, our one year follow-up of Rai stage III and IV CLL patients showed no significant change in the number of peripheral CD4+ and CD8+ T cells. One possible reason is that only a partial number of patients were in complete remission after one year treatment, or alternatively, that the notable reduction in peripheral lymphocytes may have altered CD4+ T cell proportions. 

This study also showed that the concentrations of IL-6, IL-17 and IL-23 were all significantly higher in CLL patients, while concentrations of TGF-β1 and IL-10 were clearly lower than controls. This suggested that the normal cytokine micro-environment, which could potentially maintain the balance of Treg and Th17 cells, was damaged in CLL. To verify this idea, we followed the number of Th17 and Treg cells in Rai stage III and IV CLL patients for 12 months (M3, M6 and M12) and observed that the frequency of Treg cells was upregulated after three months treatment (M3 vs. M0, p<0.05). However, there was no statistically significant difference in the number of cells at other treatment time points. Thus, the results of our follow-up of Treg cells did not support our primary finding that peripheral CD4+CD25+Foxp3+Treg cells were reduced in CLL patents. Other studies of CLL patients have shown great variability in the percentage of Treg cells.^12^ Beyer et al. reported elevated frequencies of Treg cells,^13^ while D'Arena G, et al. reported a lower percentage of Treg cells in CLL patients, but a higher absolute number.^14^ The disagreement between our results and other studies may be due to several reasons including statistical methods (absolute count vs. relative proportion), race, and unbalanced autoimmune function after short term treatment. We plan to continue our follow-up study utilizing a larger population and a longer treatment term.

Reports of Th17 cells have also been contradictory. Jadidi-Niaragh et al. showed a lower frequency of Th17 cells in CLL patients,^15^ but Jain et al. reported that the frequency of Th17 cells in peripheral blood and spleen cell suspensions was higher in patients with CLL.^16^ In our study, there was no significant change in Th17 cells after three months treatment, but Th17 cell frequency gradually declined as patients continued to receive treatment. This result is in accordance with our primary result, and further suggested that the frequency of Th17 cells in patients with CLL was initially upregulated, followed by down regulation after treatment.

Our results indicate that Th17 cells may play a key role in the CLL pathogenesis and progression, but the signaling pathways associated with these effects remain unclear. A recent study suggested that Th17 cells exert an anti-viral effect via activation of TGF-β and RORγt/RORα pathways.^17^ Furthermore, the anti-tumor effect of Th17 may be related to the CXCL2 induction which was shown to occur downstream from IL-17RA-dependent induction of IL-1β.^18^

In conclusion, we found that patients with CLL in Sichuan province display a CD4+ cell-mediated immune disorder. Th17 cells appear to be an important mediator of disease progression and require further study. Treg cell counts were decreased in the untreated patients and additional follow-up is needed to verify the role of Treg cells in CLL.

## Notes

***Source of Funding:*** This study was supported by the National Natural Science Foundation of China (Grant no. 81201343).

## Notes

***Author’s contribution: ***Yongqian Jia, Qiu Niu and Dijiao Tang designed the study. Dijiao Tang collected the data and wrote draft and final manuscript. The other authors enrolled the patients and collected the data. All authors approved the final version to be submitted.
